# Molecular detection of piroplasms, *Anaplasma*, and *Ehrlichia* species in Kazakhstan

**DOI:** 10.3389/fvets.2025.1533589

**Published:** 2025-02-03

**Authors:** Weixin Zeng, Zhumanov Kairat, Madina Awulibieer, Sansyzbay Abylay, Khizat Serik, Meihua Yang, Yuanzhi Wang, Wurelihazi Hazihan

**Affiliations:** ^1^College of Animal Science and Technology, Shihezi University, Shihezi, China; ^2^Kazakh National Agrarian Research University, Almaty, Kazakhstan; ^3^Department of Forest, College of Agriculture, Shihezi University, Shihezi, China; ^4^NHC Key Laboratory of Prevention and Treatment of Central Asia High Incidence Diseases, School of Medicine, Shihezi University, Shihezi, China

**Keywords:** ticks, tick-borne pathogens, morphological identification, genotype, Kazakhstan

## Abstract

Tick-borne pathogens (TBPs) are a global public health issue. However, there have been few reports on the prevalence of piroplasms, *Anaplasma*, and *Ehrlichia* in Kazakhstan. To understand the distribution of piroplasms, *Anaplasma*, and *Ehrlichia* pathogens carried by ticks in Kazakhstan, a total of 10,461 ticks were collected from natural hosts (e.g., cattle, sheep, and horses) in six oblasts in eastern, southern, and western Kazakhstan between 2022 and 2024. After morphological identification, 272 representative ticks were further used for species-level detection and partial genotyping analysis of TBPs. Two *Babesia* species (*Babesia occultans* and *Babesia caballi*), four *Theileria* species (*Theileria orientalis*, *Theileria equi*, *Theileria annulata*, and *Theileria ovis*), two *Anaplasma* species (*Anaplasma phagocytophilum* and *Anaplasma ovis*), and three *Ehrlichia* species were detected. Furthermore, genotype B of *B. caballi*, genotype 1 (Chitose) of *T. orientalis*, and genotype A of *T. equi* were confirmed. For the first time, *A. phagocytophilum*, three phylogeny-independent *Ehrlichia* spp., genotype B of *B. caballi*, and genotype A of *T. equi* were found in Kazakhstan. These findings expand our understanding of the geographical distribution of piroplasms, *Anaplasma*, and *Ehrlichia* in Central Asia.

## 1 Introduction

As hematophagous ectoparasites, ticks can transmit a variety of zoonoses (1, 2). *Babesia*, *Theileria*, *Anaplasma*, and *Ehrlichia* are tick-borne pathogens (TBPs) that infect a variety of reservoir animals, including domestic animals (e.g., cattle, sheep, and horses) and wildlife. *Babesia* and *Theileria*, belonging to the order of Piroplasmida, can cause babesiosis and theileriosis in animals and occasionally in humans. To date, more than 50 species piroplasmida in domestic and wild species have been reported (3–7). *Anaplasma* and *Ehrlichia* belong to the order of Anaplasmataceae, with at least eight validated *Anaplasma* species and eight identified *Ehrlichia* species.

Kazakhstan, which covers 2,724,900 km^2^ in Central Asia, is listed as the ninth largest country in the world. TBPs play a vital role in veterinary medicine and public health. Some TBPs, such as Crimean–Congo hemorrhagic fever virus, spotted fever rickettsia, and tick-borne encephalitis virus, have already been reported in Kazakhstan (8–10). However, there have been few reports on the prevalence of piroplasms, *Anaplasma*, and *Ehrlichia* in Kazakhstan. In the present study, we aimed to detect *Babesia*, *Theileria*, *Anaplasma*, and *Ehrlichia* in ticks parasitizing cattle, horses, sheep, pet dogs, and hens in the east, south, and west regions of Kazakhstan.

## 2 Materials and methods

### 2.1 Tick sampling

From March to May in 2022, 2023, and 2024, an extensive tick sampling program was conducted in six oblasts of Kazakhstan (Jetysu, Jambyl, Almaty, Turkistan, Kyzylorda, and Aktobe oblasts). Parasitic ticks were collected from the whole body of cattle, horses, sheep, pet dogs, and hens.

### 2.2 Identification of ticks

Morphological identification was conducted on all of the collected ticks (*n* = 10,461) (11, 12). The ticks’ morphological features were examined under a stereoscopic dissecting microscope. After the morphological identification, 272 representative ticks were selected for DNA extraction using the TIANamp Genomic DNA Kit (TIANGEN, Beijing, China) following the manufacturer’s instructions. The obtained genomic DNAs from these representative ticks were then subjected to molecular identification using the fragments of cytochrome c oxidase subunit 1 (*cox1*) and *16S rDNA* genes (Appendix Table 1).

### 2.3 Isolation and identification of piroplasms, *Anaplasma*, and *Ehrlichia* pathogens

The detection of piroplasms, *Anaplasma*, and *Ehrlichia* was performed by nested PCR. We used the universal primers of *18S rRNA* gene to detect *Theileria* and *Babesia*. *Anaplama* and *Ehrlichia* were detected using a partial *16S rRNA* gene (Appendix Table 1). The DNAs of *Theileria equi*, *Babesia caballi*, *Anaplasma ovis* and *Ehrlichia* spp. in our laboratory were used as positive controls (13–15). Double-distilled water was used as a negative control. The amplified products were cloned into the pGEM-T Easy Vector (TransGen Biotech, Beijing, China) according to the manufacturer’s instructions and then subjected to Sanger sequencing. To gain insights into the evolutionary relationships and taxonomic affiliations of the identified pathogens, the obtained nucleotide sequences were queried against the GenBank database using BLASTn.^1^ Additionally, phylogenetic trees were constructed employing the Neighbor-Joining (NJ) algorithm within MEGA11 software (bootstrap replicates 1,000).

## 3 Results

**Figure 1:**
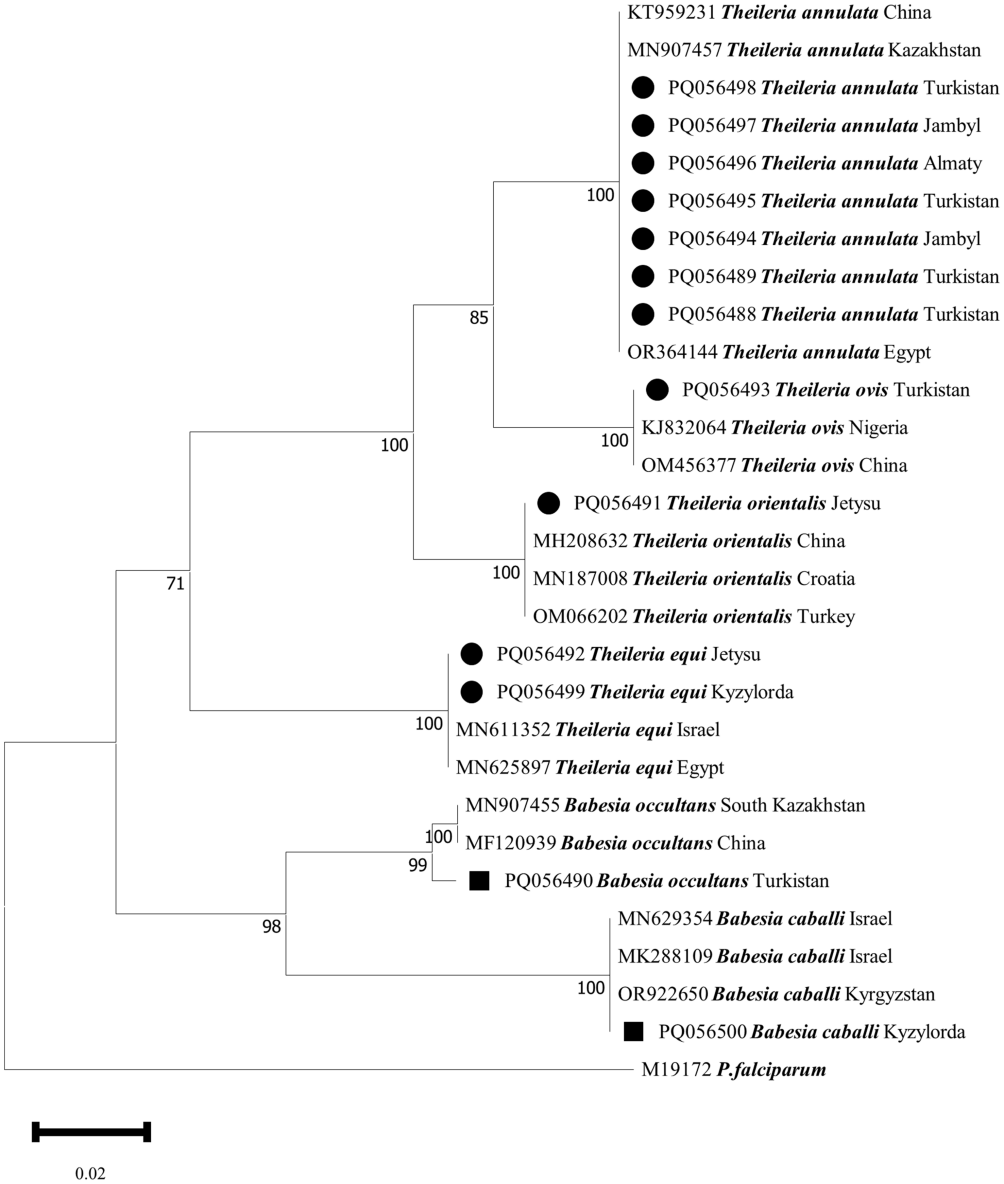
Phylogenetic analysis of *Babesia* spp. and *Theileria* spp. in ticks collected in Kazakhstan. The tree was constructed using the Neighbor-Joining (NJ; bootstrap replicates: 1000) method based on the sequence data for *18S rRNA* genes with MEGA11.0. The sequences of the *Theileria* species from ticks obtained in this study are indicated by solid circles (●), and those of the *Babesia* species are indicated by solid squares (■).

**Figure 2:**
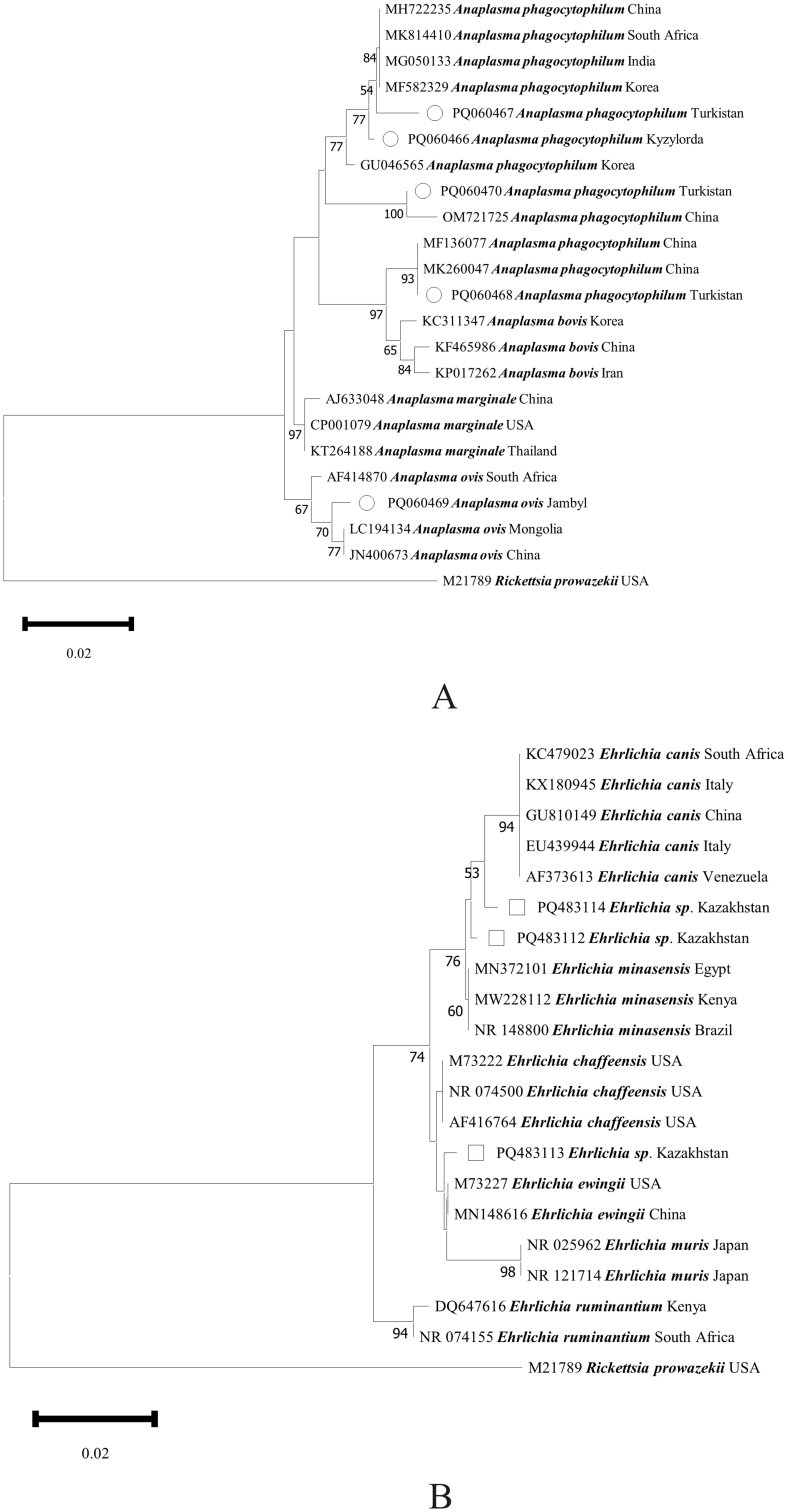
Phylogenetic analysis of *Anaplasma* spp. **(A)** and *Ehrlichia* spp. **(B)** in ticks collected in Kazakhstan. The tree was constructed using the Neighbor-Joining (NJ; bootstrap replicates: 1000) method based on the sequence data for *16S rRNA* genes with MEGA11.0. *Anaplasma* spp. are indicated by hollow circles (○), and *Ehrlichia* spp. are indicated by hollow squares (□).

Five tick species belonging to three genera were identified from 272 representative ticks, namely *Hyalomma scupense* (*n* = 126), *Hyalomma asiaticum* (*n* = 34), *Hyalomma anatolicum* (*n* = 75), *Rhipicephalus turanicus* (*n* = 24), and *Argas persicus* (*n* = 13). A total of 11 TBPs were detected: *Theileria orientalis*, *Theileria equi*, *Theileria ovis*, *Theileria annulata*, *Babesia occultans*, *Babesia caballi*, *Anaplasma phagocytophilum*, *Anaplasma ovis*, and three phylogeny-independent *Ehrlichia* spp. (shown in Figures 1, 2 and Table 1). The information on the pathogens’ sequence similarities and their geographical distribution in this study is presented in Appendix Table 2.

**Table 1:** Detection of piroplasms, *Anaplasma*, and *Ehrlichia* spp. in ticks sampled from six oblasts of Kazakhstan.

Scheme					Positive ticks per pathogen species (%)			
Oblast	County	No	Tick	Host	*Babesia* species	*Theileria* species	*Anaplasma* species	*Ehrlichia* species
Jetysu	Karabulak	15	*Hyalomma scupense*	Cattle	0	0	0	0
	Balpyk Bi	30	*Hy. scupense*	Horse	0	*T. orientalis*, 1 (3.33%) *T. equi*, 1 (3.33%)	0	0
Turkistan	Sayram	24	*Hy. aisaticum*	Sheep	*B. occultans*, 1 (4.17%)	*T. ovis*, 1 (4.17%)	0	0
		14	*Rhipicephalus turanicus*		0	0	*A. phagocytophilum*, 1 (7.14%)	0
		13	*Argas persicus*	Hen	0	0	*A. phagocytophilum*, 1 (7.69%)	0
	Tulkibas	15	*Hy. anatolicum*	Cattle	0	*T. annulata,* 1 (6.67%)	0	0
	Saryagash	13	*Hy. anatolicum*	Sheep	0	*T. annulata,* 1 (7.69%)	0	0
	Kzygurt	17	*Hy. anatolicum*	Cattle	0	*T. annulata*, 1 (5.88%)	*A. phagocytophilum*, 1 (5.88%)	0
		12	*Hy. scupense*		0	*T. annulata*, 1 (8.33%)	0	*Ehrlichia* spp., 1 (8.33%)
Kyzylorda	Shieli	35	*Hy. scupense*	Cattle	0	*T. equi*, 1 (2.86%)	*A. phagocytophilum*, 1 (2.86%)	0
	Aral	10	*Hy. aisaticum*	Cattle	*B. caballi*, 1 (10.00%)	0	0	0
		8	*Hy. scupense*		0	0	0	0
	Karmakshy	15	*Hy. scupense*	Cattle	0	0	0	0
Jambyl	Moiynkum	15	*Hy. anatolicum*	Cattle	0	*T. annulata*, 2 (13.33%)	*A. ovis*, 1 (6.67%)	0
Almaty	Uzynagash	15	*Hy. anatolicum*	Cattle	0	*T. annulata*, 1 (6.67%)	0	0
	-	10	*Rh. turanicus*	Dog	0	0	0	*Ehrlichia* spp., 1 (10.00%)
Aktobe	Khromtau	11	*Hy. scupense*	Horse	0	0	0	*Ehrlichia* spp., 1 (9.09%)

**Figure 3:**
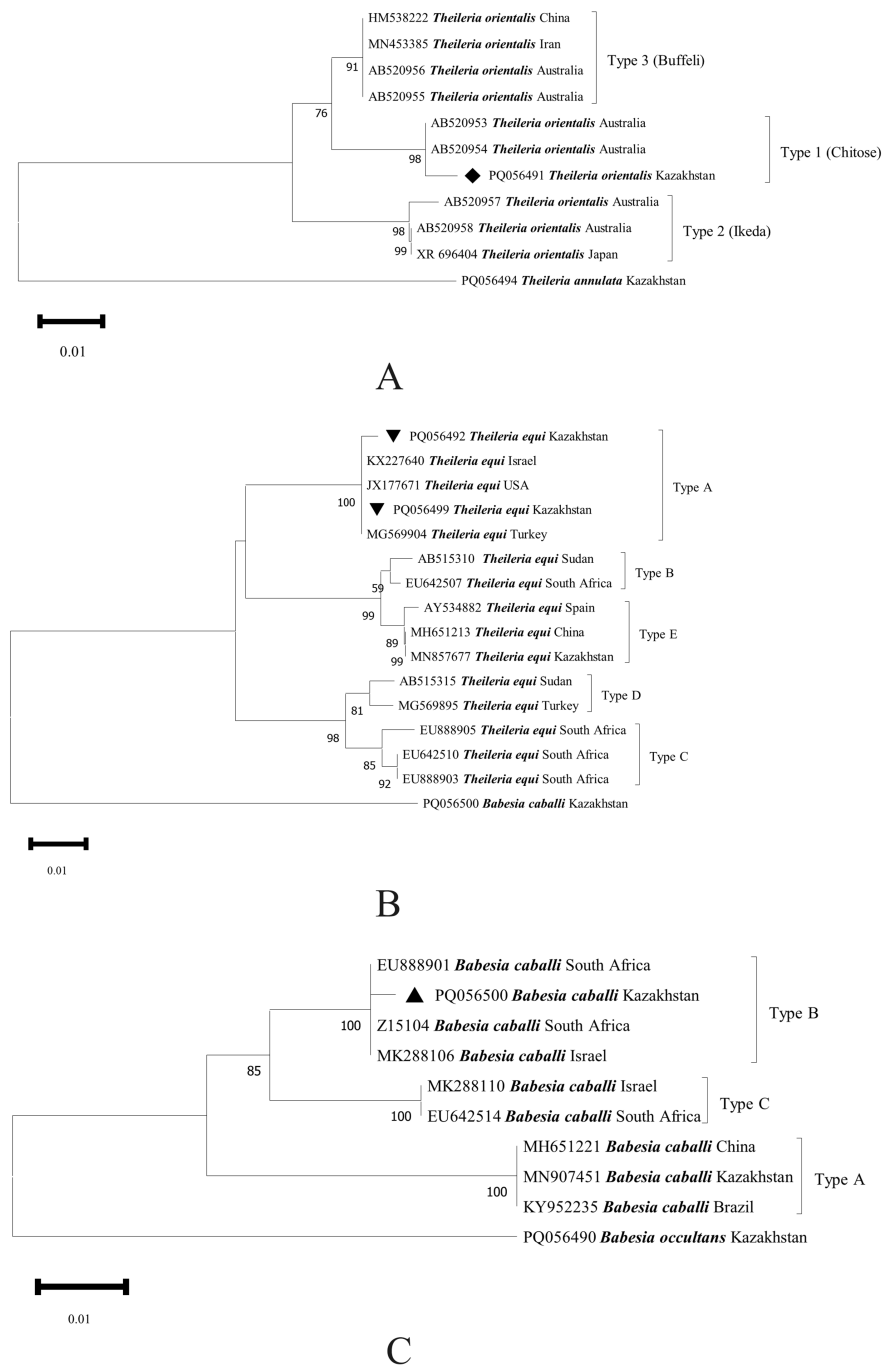
Phylogenetic tree of Theileria *orientalis* **(A)**, *T. equi* **(B)**, and *B. caballi* **(C)** genotypes inferred from the partial sequences of the *18S rRNA* gene. The sequences of *T. orientalis* obtained in this study are indicated by solid diamonds (◆), those of *T. equi* are indicated by inverted triangles (▼), and those of *B. caballi* are indicated by solid triangles (▲).

Furthermore, *T. orientalis* genotype 1 (Chitose) (PQ056491), *T. equi* genotype A (PQ056492, PQ056499), and *B. caballi* genotype B (PQ056500) were confirmed (Figure 3), which were clustered with those from Australia (AB520953), the United States (JX177671), and South Africa (Z15104), respectively.

## 4 Discussion

Kazakhstan is located in Central Asia, bordered by countries such as China, Russia, Kyrgyzstan, and Turkmenistan. International trade of domestic animals and their products is common. In this study, two *Babesia* species, four *Theileria* species, two *Anaplasma* species, and three independent *Ehrlichia* species were molecularly identified in hard ticks collected in six oblasts of southern and western Kazakhstan. Piroplasms, *Anaplasma*, and *Ehrlichia* are tick-borne pathogens of economically and medically important diseases (16–18). Domestic and wild animals play the roles of reservoirs, carriers, and disseminators in the epidemiology of many tick-borne pathogens. When vertebrates become infected, they may develop babesiosis, theileriosis, anaplasmosis, and ehrlichiosis (18–20). These diseases restrict livestock production and even impact public health in developing countries, including Kazakhstan.

In summary, the prevalence rate of both *B. occultans* and *B. caballi* in *Hy. asiaticum* stands at 2.93%. In *Hy. scupense*, the prevalence rate for *T. orientalis* and *T. annulata* is 0.79%, whereas that for *T. equi* and *Ehrlichia* spp. is 1.59%. In *Rhipicephalus turanicus*, the prevalence rate for both *A. phagocytophilum* and *Ehrlichia* spp. is 4.17%. In *Hy. anatolicum*, the prevalence rate for *A. phagocytophilum* and *A. ovis* is 1.33%, with *T. annulata* having a prevalence rate of 8.00%. And in *Argas persicus*, the prevalence rate for *A. phagocytophilum* is 7.69%. Furthermore, genotypes 1 (Chitose) of *T. orientalis*, genotype A of *T. equi*, and genotype B of *B. caballi* were confirmed.

Previously, *T. annulata* and *B. caballi* were detected in hard ticks in Turkistan oblast (South Kazakhstan) (5). *T. annulata*, *T. orientalis*, *B. bigemina*, *B. major*, and *B. occultans* were detected in bovine blood from Turkistan and Jambyl oblasts, and genotypes 1 (Chitose) and 3 (Buffeli) of *T. orientalis* were further confirmed (21). *B. caballi*, *T. annulata*, *T. equi*, *B. occultans*, and *T. ovis* were detected in hard ticks in Almaty and Turkistan oblasts, and *T. equi* genotype E and *B. caballi* genotype A were also confirmed (22). In this study, *Babesia* species (*B. caballi* genotype B) and two *Theileria* species (*T. orientalis* genotype 1 [Chitose] and *T. equi* genotype A) were found in Kyzylorda and Jetysu oblasts (southern and eastern Kazakhstan) for the first time. These findings indicate more genetic diversity among piroplasms in Kazakhstan.

Only three *Anaplasma* species were previously reported in bovine blood samples in Kazakhstan, namely *A. ovis* in Turkistan oblast, *A. marginale in* Kyzylorda oblast, and *A. centrale* in North Kazakhstan oblast (GenBank accession nos.: PQ133423, PQ038050, and PQ038051). In 2015, our team detected *A. phagocytophilum* in *Hy. asiaticum* ticks in Almaty oblast (KU723458). Here, *A. phagocytophilum* was first screened out in Turkistan and Kyzylorda oblasts. In Kazakhstan’s neighboring countries, *A. ovis* strains were detected in *Hy. marginatum*, *Rh. turanicus*, and *Dermacentor* spp. ticks in Kyrgyzstan and clustered with those in China (MG869525) (23). *A. ovis* strains in *Rh. turanicus* and *Hy. anatolicum* ticks were detected in China and clustered with those in Tunisia (KY659323), Pakistan (MT311202), Italy (GQ130291), and Turkey (OQ167969) (24). In this study, *A. ovis* was detected in *Hy. anatolicum* in Jambyl oblast, and it showed an independent clade, although it is comparatively close to those found in sheep blood in Mongolia (LC194134) and China (JN400673). *A. phagocytophilum* was commonly detected in hard ticks. Meanwhile, it was rarely found in soft ticks, including *A. lahorensis*, *A. japonicus*, and *A. persicus* in China (GenBank accession nos.: MG668811, MN795629, and ON807566). Here, *A. phagocytophilum* strains were detected in *A. persicus* and hard ticks (e.g., *Hy. anatolicum*, *Hy. scupense*, and *Rh. turanicus*), and they showed high genetic diversity, especially in the 74–84 bp fragment with U02528 as the original sequence for comparison (Appendix Table 3). To date, 14 genotypes have been reported in *A. phagocytophilum.* Given the lack of data on *Anaplasma* in Central Asia, more investigation on *Anaplasma* should be done in the future.

To date, *Ehrlichia* includes eight validated species, such as *E. chaffeensis*, *E. ewingii*, and *E. canis*, along with numerous indeterminate species reported. Previously, multiple indeterminate *Ehrlichia* strains were detected in *Amblyomma longirostre*, *Am. cajennense*, *Am. romitii*, *Rh. microplus*, and *Rh. pusillus* ticks (25, 26). In the present study, three phylogeny-independent *Ehrlichia* strains were detected. One strain originated from pet dog ticks (*Rh. turanicus*) from a veterinary clinical hospital in Almaty oblast, the second one was from cattle ticks (*Hy. scupense*) in Turkistan oblast, and the third one was from horse ticks (*Hy. scupense*) in Aktobe oblast. The discovery and distribution of *Ehrlichia* species are closely related to their natural hosts and geographical locations. Expanding the sampling to include more tick species, domestic animals, wildlife, and additional sites will be important for future investigations of *Ehrlichia* species in Central Asia.

Interestingly, we detected *A. ovis*, *B. caballi*, and *T. equi* in ticks collected from cattle, despite the fact that *A. ovis* is generally considered to be primarily detected in sheep (27, 28), while *B. caballi* and *T. equi* are typically found in horses or equines (29, 30). Domestic animals such as cattle, horses, sheep, and camels are natural hosts for species such as *Hy. asiaticum*, *Hy. scupense*, *Hy. anatolicum*, and *Rh. turanicus* (31, 32). Occasionally, these animals may also host *Argas persicus* (23). According to reports, ticks infected with *T. equi* and *A. ovis* are unable to directly transmit the pathogens to the offspring, while ticks infected with *B. caballi* can directly pass it on to the next tick generation (6, 33, 34). Moreover, *A. ovis* does not have strict host specificity and has been detected in cattle in addition to sheep and goats (35–37), which is consistent with our findings. In this study, *T. equi* and *A. ovis* were detected in ticks collected from cattle. This could be due to the ticks migrating to cattle after feeding on infected animals. As for *B. caballi*, it may be carried by the ticks or the cattle themselves. Regarding these findings, future research may delve deeper into aspects such as expanding the range of hosts for tick – borne diseases, the interaction between hosts and pathogens, and the migration of tick vectors and hosts. Therefore, we speculate that cross-species transmission may have occurred as ticks fed on the blood of different hosts. Such cross-species transmission has the potential to cause unknown diseases or symptoms in new hosts, posing a potential threat to public health and animal welfare. Furthermore, these finding underscores the need for further research into the host range and transmission patterns of these pathogens to better understand their distribution and epidemiology in nature.

In this study, although we could not determine whether these TBPs originated from the engorged ticks or their hosts, we still believe that multiple piroplasms, *Anaplasmas*, and *Ehrlichia* exist in Kazakhstan. Due to the lack of more data in Kazakhstan and its neighboring countries (especially in Central Asian countries), the taxonomy of TBPs at the level of species and genotype needs further research.

## 5 Conclusion

Two species of *Babesia* (*B. occultans* and *B. caballi*), four species of *Theileria* (*T. annulata*, *T. ovis*, *T. equi*, and *T. orientalis*), two species of *Anaplasma* (*A. phagocytophilum* and *A. ovis*), and three phylogeny-independent *Ehrlichia* species were detected in 259 hard ticks and 13 soft ticks in six oblasts in Kazakhstan. The genotype 1 (Chitose) of *T. orientalis*, genotype B of *B. caballi*, and genotype A of *T. equi* were further confirmed. These findings expand the geographical distribution and knowledge of TBPs in Central Asia, especially in Kazakhstan.

## Data Availability

The datasets presented in this study can be found in online repositories. The names of the repository/repositories and accession number(s) can be found at: https://www.ncbi.nlm.nih.gov/genbank/ (*Babesia caballi* 18S rRNA: PQ056500; *Babesia occultans* 18S rRNA: PQ056490; *Theileria equi* 18S rRNA: PQ056499; *Theileria annulata* 18S rRNA: PQ056488-89; PQ056494-98; *Theileria ovis* 18S rRNA: PQ056493; *Theileria orientalis* 18S rRNA: PQ056491; *Anaplasma phagocytophilum* 16S rRNA: PQ060466-68; PQ060470; *Anaplasma ovis* 16S rRNA: PQ060469; *Ehrlichia* species 16S rRNA: PQ483112-14).

## Footnotes

^1^ https://blast.ncbi.nlm.nih.gov/Blast.cgi

## Supplementary material

### APPENDIX TABLE 1

PCR protocol for the detection of ticks specimens and pathogens.

### APPENDIX TABLE 2

Data of piroplasm, *Anaplasma* and *Ehrlichia* species identified in ticks including 18S rRNA and 16S rRNA genes with NCBI BLAST maximum identity percentages.

### APPEND IX TABLE 3

*A. phagocytophilum* genetic variants of 16S rRNA gene fragments.
