# Supplementary material for: Molecular detection of piroplasms, Anaplasma, and Ehrlichia species in Kazakhstan
Source: Front Vet Sci. 2025 Feb 3;12:1533589. doi: 10.3389/fvets.2025.1533589 (PMC11831815; doi:10.3389/fvets.2025.1533589)
Supplement: Supplementary file 1 [file Data_Sheet_1.ZIP › Appendix Table 1.docx]

**PCR protocol for the detection of tick specimens and pathogens, Kazakhstan.**

The PCR equipment was a Mastercycler X50s thermal cycler, Eppendorf, Hamburg, Germany.

**PCR amplification of *16S rDNA* , *cox1*, *18S rRNA* , *16S rRNA* gene sequences from tick specimens and pathogens**

Each reaction consisted of 1 μL of tick genomic DNA (50 ng) and 25 μL of a PCR mix containing 16.5 μL of ultrapure water, 2.4 μL of 10× PCR buffer, 2 μL of dNTPs (10 mmol/L), 1.5 μL of the DNA template (50ng/μL), 1 μL of each primer (20 μmol/L) and 0.6 μL of Taq DNA polymerase (Taq DNA Polymerase, GDSBio, Guangzhou, China).

*16S rDNA* gene of the cycling conditions consisted of an initial 5-min denaturation at 94°C, followed by 37 cycles at 92°C for 30 s, 54°C for 30 s, and 72°C for 30 s, with a final extension at 72°C for 8 min

*cox1* gene of the cycling conditions consisted of an initial 5-min denaturation at 94°C, followed by 37 cycles at 94°C for 30 s, 46°C for 30 s, and 72°C for 90 s, with a final extension at 72°C for

8 min

*18S rRNA* gene is amplified by nested PCR. The first of the program is the conditions consisted of an initial 5-min denaturation at 95°C, followed by 35 cycles at 94°C for 45 s, 55°C for 45 s, and 72°C for 60 s, with a final extension at 72°C for 7 min. the next program same as the first program.

*16S rRNA* gene is amplified by nested PCR. The first of the program is the conditions consisted of an initial 5-min denaturation at 95°C, followed by 40 cycles at 94°C for 20 s, 56°C for 30 s, and 72°C for 40 s, with a final extension at 72°C for 5 min. the next program same as the first program.

| Target organism | Gene | Primer | Sequence (5’-3’) | Fragment size (bp) | References |
| --- | --- | --- | --- | --- | --- |
| Tick species | *16S rDNA* | 16s-F  16s-R | CTGCTCAATGATTTTTTAAATTGCTGTGG  CCGGTCTGAACTCAGATCAAGT | 460 | (Black et al. 1994) |
|  | *cox1* | Tick-*cox1*-F  Tick-*cox1-*R | GGTCAACAAATCATAAAGATATTGG | 710 | (Lv et al. 2014) |
|  |  |  | TAAACTTCAGGGTGACCAAAAAATCA |  |  |
| Piroplasm | *18S rRNA* | PIRO-F1  PIRO-R1  PIRO-F2  PIRO-R2 | AATACCCAATCCTGACACAGGG  TTAAATACGAATGCCCCCAAC  GACACAGGGAGGTAGTGACAAGA  CCCAACTGCTCCTATTAACCATTAC | 400 | (Song et al. 2018) |
|  |  | Bab-F1 | AATTACCCAATCCTGACACAGG | 400 | (Wei et al. 2001; Liu et al. 2016) |
|  |  | Bab-R1  Bab-F2  Bab-R2 | TTTCGCAGTAGTTCGTCTTTAACA  GACAAGAAATAACAATACRGGGC  CTAAGAATTTCACCTCTGACAGT |  |  |
| *Anaplasma* /*Ehrlichia* species | *16S rRNA* | OUT-F  OUT-R | TTGAGAGTTTGATCCTGGCTCAGAACG  CACCTCTACACTAGGAATTCCGCTATC | 653 | (Zeng et al. 2022) |
|  |  |  |  | 389 |  |
|  |  | IN-F | GTCGAACGGATTATTCTTTATAGCTTG |  |  |
|  |  | IN-R | TATAGGTACCGTCATTATCTTCCCTAC |  |  |

**Reference**

Black, W. C., 4th, & Piesman, J. (1994). Phylogeny of hard- and soft-tick taxa (Acari: Ixodida) based on mitochondrial 16S rDNA sequences. Proceedings of the National Academy of Sciences of the United States of America, 91(21), 10034–10038. https://doi.org/[10.1073/pnas.91.21.10034](https://doi.org/10.1073/pnas.91.21.10034)

Lv, J., Wu, S., Zhang, Y., Chen, Y., Feng, C., Yuan, X., Jia, G., Deng, J., Wang, C., Wang, Q., Mei, L., & Lin, X. (2014). Assessment of four DNA fragments (COI, 16S rDNA, ITS2, 12S rDNA) for species identification of the Ixodida (Acari: Ixodida). Parasites Vectors, 7, 93. https://doi.org/[10.1186/1756-3305-7-93](https://doi.org/10.1186/1756-3305-7-93)

Song, R., Wang, Q., Guo, F., Liu, X., Song, S., Chen, C., Tu, C., Wureli, H., & Wang, Y. (2018). Detection of Babesia spp., Theileria spp. and Anaplasma ovis in Border Regions, northwestern China. Transbound Emerg Dis, 65(6), 1537–1544. https://doi.org/[10.1111/tbed.12894](https://doi.org/10.1111/tbed.12894)

Wei, Q., Tsuji, M., Zamoto, A., Kohsaki, M., Matsui, T., Shiota, T., Telford, S. R., 3rd, & Ishihara, C. (2001). Human babesiosis in Japan: isolation of Babesia microti-like parasites from an asymptomatic transfusion donor and from a rodent from an area where babesiosis is endemic. J Clin Microbiol, 39(6), 2178–2183. https://doi.org/[10.1128/JCM.39.6.2178-2183.2001](https://doi.org/10.1128/JCM.39.6.2178-2183.2001)

Liu, J., Yang, J., Guan, G., Liu, A., Wang, B., Luo, J., & Yin, H. (2016). Molecular detection and identification of piroplasms in sika deer (Cervus nippon) from Jilin Province, China. Parasites Vectors, 9, 156. https://doi.org/[10.1186/s13071-016-1435-3](https://doi.org/10.1186/s13071-016-1435-3)

Zeng, W., Li, Z., Jiang, T., Cheng, D., Yang, L., Hang, T., Duan, L., Zhu, D., Fang, Y., & Zhang, Y. (2022). Identification of Bacterial Communities and Tick-Borne Pathogens in Haemaphysalis spp. Collected from Shanghai, China. Trop Med Infect Dis, 7(12), 413. https://doi.org/[10.3390/tropicalmed7120413](https://doi.org/10.3390/tropicalmed7120413)
