# Supplementary material for: Molecular detection of piroplasms, Anaplasma, and Ehrlichia species in Kazakhstan
Source: Front Vet Sci. 2025 Feb 3;12:1533589. doi: 10.3389/fvets.2025.1533589 (PMC11831815; doi:10.3389/fvets.2025.1533589)
Supplement: Supplementary file 1 [file Data_Sheet_1.ZIP › Appendix Table 2 .docx]

| Pathogens | Oblasts | County | Tick species | *18S rRNA* (NCBI BLAST maximum identity) | *16S rRNA* (NCBI BLAST maximum identity) |
| --- | --- | --- | --- | --- | --- |
| *T. orientalis* | Jetysu | [Balpyk Bi](https://en.wikipedia.org/wiki/Balpyk_Bi) | *H. scupense* | 99.48 % with *T . orientalis* (LC602477) | NA |
| *T. ovis* | Turkistan | Sayram | *H. aisaticum* | 100 % with *T. ovis* (KJ832064) | NA |
| *T. equi* | Jetysu | [Balpyk Bi](https://en.wikipedia.org/wiki/Balpyk_Bi) | *H. scupense* | 99.50 % with *T. equi* (MT463608) | NA |
|  | [Kyzylorda](https://www.bing.com/ck/a?!&&p=529b0ae3f78cdb7fJmltdHM9MTcxOTE4NzIwMCZpZ3VpZD0zYTU4ZjY5OC00NzkwLTZhYWEtMzBlNi1lNTY5NDZlZTZiZmQmaW5zaWQ9NTI2MA&ptn=3&ver=2&hsh=3&fclid=3a58f698-4790-6aaa-30e6-e56946ee6bfd&psq=%e5%85%8b%e5%ad%9c%e5%8b%92%e5%a5%a5%e5%b0%94%e8%be%be%e5%b7%9e&u=a1aHR0cHM6Ly9rYXpha2hzdGFuLnRyYXZlbC96aC1DTi93aGVyZS10by1nby9yZWdpb24vMTMva3l6eWxvcmRhLXByb3ZpbmNl&ntb=1) | Shieli | *H. scupense* | 99.53 % with *T. equi* (OR960553) | NA |
| *T. annulata* | Turkistan | Tulkibas | *H. anatolicum* | 98.42 % with *T . annulata* (MK182996) | NA |
|  |  | [Saryagash](https://en.wikipedia.org/wiki/Saryagash_District) | *H. anatolicum* | 99.77 % with *T . annulata* (MW046053) | NA |
|  |  | Kzygurt | *H. anatolicum* | 99.49 % with *T . annulata* ([KT367874](https://www.ncbi.nlm.nih.gov/nucleotide/KT367874.1?report=genbank&log$=nucltop&blast_rank=2&RID=EZVGVDEX016)) | NA |
|  |  |  | *H. scupense* | 100 % with *T . annulata* ([KT367874](https://www.ncbi.nlm.nih.gov/nucleotide/KT367874.1?report=genbank&log$=nucltop&blast_rank=2&RID=EZVGVDEX016)) | NA |
|  | Jambyl | Moiynkum | *H. anatolicum* | 99.29 % with *T . annulata* (MK182996) | NA |
|  | Almaty | Uzynagash | *H. anatolicum* | 100 % with *T . annulata* (MK182996) | NA |
| *B.occultans* | Turkistan | Sayram | *H. aisaticum* | 99.46 % with *B.occultans* (PP668148) | NA |
| *B. caballi* | [Kyzylorda](https://www.bing.com/ck/a?!&&p=529b0ae3f78cdb7fJmltdHM9MTcxOTE4NzIwMCZpZ3VpZD0zYTU4ZjY5OC00NzkwLTZhYWEtMzBlNi1lNTY5NDZlZTZiZmQmaW5zaWQ9NTI2MA&ptn=3&ver=2&hsh=3&fclid=3a58f698-4790-6aaa-30e6-e56946ee6bfd&psq=%e5%85%8b%e5%ad%9c%e5%8b%92%e5%a5%a5%e5%b0%94%e8%be%be%e5%b7%9e&u=a1aHR0cHM6Ly9rYXpha2hzdGFuLnRyYXZlbC96aC1DTi93aGVyZS10by1nby9yZWdpb24vMTMva3l6eWxvcmRhLXByb3ZpbmNl&ntb=1) | Aral | *H.aisaticum* | 100 % with *B. caballi* (Z15104) | NA |
| *A. ovis* | Jambyl | Moiynkum | *H. anatolicum* | NA | 98.52 % with *A. ovis* (OL826840) |
| *A.phagocytophilum* | Turkistan | Sayram | *R. turanicus* | NA | 99.75 % with *A.phagocytophilum* (MK260047) |
|  |  |  | *A. persicus* | NA | 98.73 % with *A.phagocytophilum* (ON795112) |
|  |  | Kzygurt | *H. anatolicum* | NA | 98.22 % with *A.phagocytophilum* (MF582329) |
|  | [Kyzylorda](https://www.bing.com/ck/a?!&&p=529b0ae3f78cdb7fJmltdHM9MTcxOTE4NzIwMCZpZ3VpZD0zYTU4ZjY5OC00NzkwLTZhYWEtMzBlNi1lNTY5NDZlZTZiZmQmaW5zaWQ9NTI2MA&ptn=3&ver=2&hsh=3&fclid=3a58f698-4790-6aaa-30e6-e56946ee6bfd&psq=%e5%85%8b%e5%ad%9c%e5%8b%92%e5%a5%a5%e5%b0%94%e8%be%be%e5%b7%9e&u=a1aHR0cHM6Ly9rYXpha2hzdGFuLnRyYXZlbC96aC1DTi93aGVyZS10by1nby9yZWdpb24vMTMva3l6eWxvcmRhLXByb3ZpbmNl&ntb=1) | Shieli | *H. scupense* | NA | 98.73 % with *A.phagocytophilum* (MF582329) |
| *Ehrlichia sp* | Turkistan | Kzygurt | *H. scupense* | NA | 98.75 % with Uncultured *Ehrlichia sp* (MF582329) |
|  | Almaty | - | *R. turanicus* | NA | 99.78 % with *Ehrlichia sp* Tibet (AF414399) |
|  | [Aktobe](https://en.wikipedia.org/wiki/Aktobe_Region) | Khromtau | *H. scupense* | NA | 98.75 % with Uncultured *Ehrlichia sp* (OM065738) |
