# Supplementary material for: Molecular detection of piroplasms, Anaplasma, and Ehrlichia species in Kazakhstan
Source: Front Vet Sci. 2025 Feb 3;12:1533589. doi: 10.3389/fvets.2025.1533589 (PMC11831815; doi:10.3389/fvets.2025.1533589)
Supplement: Supplementary file 1 [file Data_Sheet_1.ZIP › Appendix Table 3.docx]

| GenBank Accession No. | The variable fragment(74-84bp)* | Host | Country | References |
| --- | --- | --- | --- | --- |
| U02521 | ATAAAGAATAG | Human | USA | Chen, S.M et al(1994). |
| PQ060470 | ATAAA**CT**A-GG | *Argas persicus* | Kazakhstan | This study |
| PQ060466 | A-A**G**A**T**GA**C**A**A** | *Hyalomma scupense* | Kazakhstan | This study |
| PQ060467 | A**CGG**A**T**-AT**GA** | *Hyalomma anatolicum* | Kazakhstan | This study |
| PQ060468 | AT**GG**-GAA**C**A**A** | *Rhipicephalus turanicus* | Kazakhstan | This study |
| GU236611 | ATAAAGAATGA | Sheep | Norway | Kybicová et al. (2009) |
| GU236538 | ATGAGGAATAG | *Capreolus capreolus* | Germany | Scharf et al. (2011) |
| GU111747 | ATAGAGAATAA | *Ixodes ricinus* | Spain | Portillo et al. (2011) |
| EU847528 | ATAGAGTATAG | Dog | Czech | Kybicová et al. (2009) |
| EU847527 | ATAAAGTATAG | Dog | Czech | Kybicová et al. (2009) |
| DQ458805 | ATGGAGGATAA | *Niviventer confucianus* | China | Zhan et al(2008) |
| AY281785 | ATAAAGGATAA | *Ixodes ricinus* | Germany | Loewenich et al(2003) |
| AY193887 | ATGAAGAATAA | *Ixodes scapularis* | USA | Courtney et al(2003) |
| AY079425 | ATAAAGATTAG | *Ixodes persulcatus* | China | Cao et al(2003) |
| AY035312 | ATAGAGAATAG | Sheep | Norway | Stuen et al(2002) |
| AF172166 | ATAAGGAATAA | House | USA | Chae et al(2000) |
| AF093789 | ATAAAGAATAA | Human | USA | Chae et al(2000) |
| AF036647 | GTAAAGAATAA | House | USA | Chae et al(2000) |

*Positions of variable nucleotides are given according to the *A. phagocytophilum* *16S rRNA* gene sequence (U02521). Nucleotides differed from this study are marked in bold

**References**

Chen, S. M., Dumler, J. S., Bakken, J. S., & Walker, D. H. (1994). Identification of a granulocytotropic Ehrlichia species as the etiologic agent of human disease.  J Clin Microbiol, 32(3), 589–595. https://doi.org/[10.1128/jcm.32.3.589-595.1994](https://doi.org/10.1128/jcm.32.3.589-595.1994)Kartashov, M. Y., Kononova, Y. V., Petrova, I. D., Tupota, N. L., Mikryukova, T. P., Ternovoi, V. A., Tishkova, F. H., & Loktev, V. B. (2020). Detection of Ehrlichia spp. and Theileria spp. in Hyalomma anatolicum ticks collected in Tajikistan. Vavilovskii Zhurnal Genet Selektsii, 24(1), 55–59. https://doi.org/[10.18699/VJ20.595](https://doi.org/10.18699/VJ20.595)

Basit, M. A., Ijaz, M., Khan, J. A., Ashraf, K., & Abbas, R. Z. (2022). Molecular Evidence and Hematological Profile of Bovines Naturally Infected with Ehrlichiosis in Southern Punjab, Pakistan. Acta Parasitol, 67(1), 72–78. <https://doi.org/10.1007/s11686-021-00433-0>

Kybicová, K., Schánilec, P., Hulínská, D., Uherková, L., Kurzová, Z., & Spejchalová, S. (2009). Detection of Anaplasma phagocytophilum and Borrelia burgdorferi sensu lato in dogs in the Czech Republic. Vector Borne Zoonotic Dis (Larchmont, N.Y.), 9(6), 655–661. https://doi.org/[10.1089/vbz.2008.0127](https://doi.org/10.1089/vbz.2008.0127)

Scharf, W., Schauer, S., Freyburger, F., Petrovec, M., Schaarschmidt-Kiener, D., Liebisch, G., Runge, M., Ganter, M., Kehl, A., Dumler, J. S., Garcia-Perez, A. L., Jensen, J., Fingerle, V., Meli, M. L., Ensser, A., Stuen, S., & von Loewenich, F. D. (2011). Distinct host species correlate with Anaplasma phagocytophilum ankA gene clusters. J Clin Microbiol, 49(3), 790–796. <https://doi.org/10.1128/JCM.02051-10>

Portillo, A., Pérez-Martínez, L., Santibáñez, S., Santibáñez, P., Palomar, A. M., & Oteo, J. A. (2011). Anaplasma spp. in wild mammals and Ixodes ricinus from the north of Spain. Vector Borne Zoonotic Dis (Larchmont, N.Y.), 11(1), 3–8. <https://doi.org/10.1089/vbz.2009.0214>

Zhan, L., CAO, W. C., de Vlas, S., Xie, S. Y., Zhang, P. H., WU, X. M., Dumler, J. S., Yang, H., Richardus, J. H., & Habbema, J. D. (2008). A newly discovered Anaplasma phagocytophilum variant in rodents from southeastern China. Vector Borne Zoonotic Dis (Larchmont, N.Y.), 8(3), 369–380. <https://doi.org/10.1089/vbz.2007.0211>

von Loewenich, F. D., Baumgarten, B. U., Schröppel, K., Geissdörfer, W., Röllinghoff, M., & Bogdan, C. (2003). High diversity of ankA sequences of Anaplasma phagocytophilum among Ixodes ricinus ticks in Germany. .J Clin Microbiol, 41(11), 5033–5040. https://doi.org/[10.1128/JCM.41.11.5033-5040.2003](https://doi.org/10.1128/JCM.41.11.5033-5040.2003)

Courtney, J. W., Dryden, R. L., Montgomery, J., Schneider, B. S., Smith, G., & Massung, R. F. (2003). Molecular characterization of Anaplasma phagocytophilum and Borrelia burgdorferi in Ixodes scapularis ticks from Pennsylvania. J Clin Microbiol, 41(4), 1569–1573. https://doi.org/10.1128/JCM.41.4.1569-1573.2003

Cao, W. C., Zhao, Q. M., Zhang, P. H., Yang, H., Wu, X. M., Wen, B. H., Zhang, X. T., & Habbema, J. D. (2003). Prevalence of Anaplasma phagocytophila and Borrelia burgdorferi in Ixodes persulcatus ticks from northeastern China. Am J Trop Med Hyg, 68(5), 547–550. https://doi.org/[10.4269/ajtmh.2003.68.547](https://doi.org/10.4269/ajtmh.2003.68.547)

Stuen, S., Van De Pol, I., Bergström, K., & Schouls, L. M. (2002). Identification of Anaplasma phagocytophila (formerly Ehrlichia phagocytophila) variants in blood from sheep in Norway. J Clin Microbiol, 40(9), 3192–3197. https://doi.org/10.1128/JCM.40.9.3192-3197.2002.

Chae, J. S., Foley, J. E., Dumler, J. S., & Madigan, J. E. (2000). Comparison of the nucleotide sequences of 16S rRNA, 444 Ep-ank, and groESL heat shock operon genes in naturally occurring Ehrlichia equi and human granulocytic ehrlichiosis agent isolates from Northern California.J Clin Microbiol, 38 (4), 1364–1369. https://doi.org/10.1128/JCM.38.4.1364-1369.2000
